# A Good Lesson

**Published:** 1881-12

**Authors:** 


### Editorial.

### A Good Lesson.

A luckless practitioner in this neighborhood has found himself placed in an unenviable position by a mistake which might happen to many others. After treating a man for a short time, word was brought to him that the patient had suddenly died. Apparently not considering it necessary to make inquiries regarding the method by which his former patient had been suddenly converted into a corpse, he forthwith sat him down and proceeded to manufacture the death certificate required by law. It was a saving of time, and the homely proverb informs us that "time is money." But that saving may prove a costly one. For it afterward transpired that the patient probably was not dead at all; at least the person buried in his stead was not the one who had been treated by the doctor,—or, in other words, the dead man was a different individual from the former sick one. There is a mystery enveloping the whole affair, which still hides the details of this interesting transformation scene, and which gives the daily press ample opportunity to guess in sensational terms as to how and why the substitution of one body for another was made. It looks like an effort to cheat an insurance company, even if no worse crime was committed. Whatever a further examination of the case may prove, it is already evident that the practitioner was at fault in not assuring himself as to the facts, before certifying to a death. It is a flimsy excuse, to say that the same thing is continually done by the ablest and surely by the busiest physicians, and that the procedure is a mere matter of form. The law requiring these certificates was framed with a view to defeat just such criminal schemes as have been partly carried out in this instance. The necessity of enforcing that law is attested by all good sense. Within the last year our efficient City Physician has especially directed the attention of the profession to it. If any one disregards it still, he must suffer in public opinion, if not otherwise; and the mistake of this one practitioner will doubtless serve as a wholesome lesson to many others.

